# Zuoqing granules attenuate ulcerative colitis via macrophage polarization modulation: involvement of the PPAR-γ/NF-κB/STAT1 signaling axis

**DOI:** 10.3389/fphar.2025.1646545

**Published:** 2025-08-11

**Authors:** Heng Deng, Ming Li, Xiaoli Fang, Kun Tang, Shuqing Xu, Rui Ding, Zilong Li

**Affiliations:** ^1^ Department of Anorectal Surgery, Second Affiliated Hospital, Anhui University of Chinese Medicine, Hefei, China; ^2^ Department of Anorectal Medicine, The First Affiliated Hospital of Anhui University of Chinese Medicine, Hefei, China

**Keywords:** Zuoqing granules, ulcerative colitis, macrophage polarization, PPAR-γ/NF-κB/STAT1 signaling, traditional Chinese medicine

## Abstract

**Background:**

Although our prior clinical study demonstrated the efficacy of Zuoqing granules (ZQGs) in treating ulcerative colitis (UC), the underlying immunomodulatory mechanisms remained unclear. This study systematically investigated ZQG’s therapeutic effects through macrophage polarization modulation and related signaling pathways using both *in vivo* and *in vitro* models.

**Methods:**

*In vivo*, dextran sulfate sodium (DSS)-induced UC rats were divided into normal control, DSS model, and ZQG treatment groups (high/medium/low doses). Colon tissues were analyzed using histopathology [hematoxylin and eosin (HE) staining], macrophage phenotyping (CD86^+^/CD206^+^ immunofluorescence and flow cytometry), signaling pathway assessment (PPAR-γ, NF-κB p65, p-NF-κB p65, and STAT1 through Western blot/qPCR), and cytokine profiling (TNF-α, IL-6, IL-10, IL-1β, and IL-4 using ELISA). *In vitro*, lipopolysaccharide (LPS)-stimulated RAW264.7 macrophages were treated with ZQG-conditioned medium and a PPAR-γ antagonist (GW9662) to validate direct effects.

**Results:**

ZQG treatment dose-dependently (1) ameliorated colonic mucosal damage, reducing histological scores by 52% compared to that in the model group, (2) modulated macrophage polarization by increasing the M2 phenotype (CD206^+^ cells, 3.25-fold increase) while decreasing M1 macrophages (CD86^+^ cells, 70% reduction), (3) upregulated PPAR-γ expression (2.0-fold increase) while suppressing NF-κB activation (43% decrease in p-NF-κB) and STAT1 signaling (48% and 40% reductions in protein and mRNA levels, respectively), and (4) rebalanced inflammatory cytokines, with 55%–62% reductions in TNF-α, IL-6, and IL-1β and 185%–210% increases in IL-10 and IL-4. *In vitro* studies further confirmed that ZQG directly shifted macrophage polarization via PPAR-γ, inhibiting M1 polarization (an effect abolished by GW9662).

**Conclusion:**

ZQG ameliorates UC by modulating macrophage plasticity through the PPAR-γ/NF-κB/STAT1 axis, a mechanism validated *in vitro* as PPAR-γ-dependent. These findings elucidate its clinical efficacy and support its use as a multi-target UC therapy.

## 1 Introduction

Ulcerative colitis (UC), a chronic inflammatory bowel disease, is characterized by relapsing and remitting inflammation of the colonic mucosa (Iannucci et al., 2025). Growing evidence suggests that dysregulated macrophage polarization plays a pivotal role in UC pathogenesis, where the imbalance between pro-inflammatory M1 and anti-inflammatory M2 phenotypes contributes to sustained mucosal damage (Creoli et al., 2025). Although biological therapies targeting specific cytokines have shown efficacy, their high cost and variable response rates highlight the need for alternative treatment strategies (Stawowczyk and Kawalec, 2018; Dharmadasa et al., 2025).

Traditional Chinese medicine (TCM) formulations, with their multi-metabolite and multi-target characteristics, offer promising therapeutic potential for complex diseases like UC (Wang M. et al., 2024). Our previous clinical study demonstrated that Zuoqing granules (ZQGs), a TCM formulation composed of Indigo Naturalis, Phellodendron bark, Sophora flavescens, Crane grass, and Sanguisorba officinalis, significantly improved clinical symptoms and endoscopic findings in patients with sigmoid UC (Li et al., 2025). Pharmacological analysis identified indirubin and matrine as key active metabolites, yet the precise immunomodulatory mechanisms underlying ZQG’s therapeutic effects remain to be elucidated. However, mechanistic studies linking TCM formulations like ZQG to specific immunomodulatory pathways remain scarce, limiting their widespread acceptance.

Recent advances have highlighted the PPAR-γ/NF-κB/STAT1 signaling axis as a critical regulator of macrophage polarization (Yan et al., 2023). PPAR-γ activation promotes M2 polarization and tissue repair, while NF-κB and STAT1 drive M1 polarization and pro-inflammatory cytokine production (Yu et al., 2019). In UC, this balance is disrupted, leading to excessive M1 activation and impaired M2-mediated resolution of inflammation (Zhuang et al., 2021). We hypothesize that ZQG may restore macrophage homeostasis through the modulation of these key pathways.

This study aims to bridge the gap between clinical observations and mechanistic understanding by investigating (i) whether ZQG modulates macrophage polarization in dextran sulfate sodium (DSS)-induced UC, (ii) the involvement of PPAR-γ/NF-κB/STAT1 signaling in ZQG’s effects, and (iii) the consequent impact on inflammatory cytokine profiles. Our findings will provide scientific validation for ZQG’s clinical application and contribute to the development of novel immunomodulatory strategies for UC.

## 2 Materials and methods

### 2.1 Animals and UC model establishment

Animals: Male Sprague–Dawley rats (6–8 weeks old, 180–220 g) were housed under standard conditions (12-h light/dark cycle, 22 °C ± 2 °C, 50%–60% humidity) with free access to food and water.

Ethics approval: All procedures were approved by the Institutional Animal Care and Use Committee of Anhui Provincial Hospital of Traditional Chinese Medicine (No.2024AH-97-1).

DSS-induced UC model: Rats received 5% DSS (MW 36–50 kDa; Hefei Linmei Biomedicals) in drinking water for 7 days to induce acute colitis (Ji et al., 2024; Li et al., 2021). The disease activity index (DAI) was scored daily based on weight loss, stool consistency, and rectal bleeding (0–4 per parameter).

Experimental groups:

1. Normal control (NC): retention enema with no DSS and saline.
2. UC model: retention enema with saline.
3. ZQG treatment: DSS model rats were treated with a retention enema containing a ZQG solution dissolved in 1 mL of saline at graded doses: high (0.3 g/kg), medium (0.2 g/kg), and low (0.1 g/kg), based on rat body weight. The 1 mL volume/rat/day refers to the total administered mixture (ZQG + saline), using a soft catheter inserted 2 cm into the rectum with the rat held in a head-down position for 1 min to ensure drug retention. Doses were calculated from clinical equivalents and adjusted for animal translation.

Treatment duration: 14 days (DSS from Day 0 to Day 7; drug administration from Day 8 to Day 21). Each experimental group consisted of eight rats. All animals survived to the study endpoint.

### 2.2 Drug preparation and administration

ZQG: ZQG was obtained from Huarun Sanjiu Pharmaceutical Co., Ltd. (Batch No. 20240654; manufacturing license: Z2024432; Hefei city, Anhui province, China) as a standardized clinical preparation. Each gram of granules contains the following botanical drug extracts (ratios based on raw botanical drug equivalents): *Indigo naturalis* (Qing Dai, derived from *Baphicacanthus cusia* (Nees) Bremek.) [Acanthaceae; *Folium et Caulis*], 0.6 g; *Phellodendron chinense* Schneid. [Rutaceae; *Cortex Phellodendri Chinensis*], 1.2 g; *Sophora flavescens* Ait. [Fabaceae; *Radix Sophorae Flavescentis*], 1.2 g; *Agrimonia pilosa* Ledeb. [Rosaceae; *Agrimoniae Herba*], 1.2 g; and *Sanguisorba officinalis* L. [Rosaceae; *Sanguisorbae Radix*], 1.2 g.

Quality control: The chemical profiling of ZQG, including HPLC quantification of active metabolites and GC-MS analysis of volatile metabolites, was previously published by our group (Li et al., 2025, doi:10.1515/jcim-2024-0435, refer to Figure 3; Li et al., 2025 for chromatograms and methodology) (Li et al., 2025).

### 2.3 Sample collection and histopathology

Tissue harvesting: Colon tissues were collected on Day 21, divided into segments for hematoxylin and eosin (HE) staining, fixed in 4% paraformaldehyde, embedded in paraffin, sectioned (4 μm), and stained using hematoxylin and eosin.

Histological scoring (0–4): the modified Nancy index: 0 = no damage; 1 = epithelial loss; 2 = mucosal erosion; 3 = ulceration <50% crypts; and 4 = ulceration >50% crypts (Rubin et al., 2025).

### 2.4 Molecular biology analyses

Quantitative PCR (qPCR) was performed using eight independent biological replicates per group. RNA was isolated using TRIzol reagent (Invitrogen, Cat. 15596026; Carlsbad, CA, United States), followed by purification using the RNeasy Mini Kit (QIAGEN, Cat. 74104; Hilden, Germany). The following primers were used: STAT1: F-5′-GCTGCCTATGATTGCTGGTTT-3′ and R-5′-TGGTTTTCCGTATGTTGTGCT-3′; PPAR-γ: F-5′-CTCCAAGAATACCAAAGTGCGA-3′ and R-5′-GCCTGATGGTTTATCCCCACA-3′; and NF-κB p65: F-5′-CCTGGAGCAAGCCATTGTC-3′ and R-5′-GGCAAGTGATTCCAAAGTCC-3′; β-actin was used as the endogenous control. The experiment was conducted under the following conditions: 95 °C for 30 s, followed by 40 cycles at 95 °C for 5 s and 60 °C for 30 s (LightCycler 96, Roche).

Western blot was performed using eight independent biological replicates per group. Proteins were extracted using RIPA lysis buffer with protease inhibitors. The primary antibodies used included PPAR-γ (1:1,000, Proteintech, Cat. 16643-1-AP; Rosemont, IL, United States), NF-κB p65 (1:1,000, CST 8242; Danvers, MA, United States), p-NF-κB p65 (1:1,000, CST 3033), STAT1 antibody (1:1,000, Abcam, Cat. ab109461; Cambridge, United Kingdom), and β-actin (1:5,000, Proteintech 20536-1-AP). The secondary antibody was an HRP-conjugated anti-rabbit IgG (1:2000, Proteintech). Detection was carried out using a chemiluminescence analyzer (Bio-Rad; Hercules, CA, United States), followed by quantification using ImageJ.

### 2.5 Macrophage polarization analysis

Immunofluorescence was performed using double staining. Frozen tissue sections were fixed in acetone, blocked with 5% BSA, and incubated with M1 markers such as anti-CD86-FITC (1:200, BD Biosciences, 553692; San Jose, CA, United States) and M2 markers including anti-CD206-APC (1:200, BD Biosciences, 561153). Nuclei were counterstained with DAPI. Imaging was conducted using a confocal microscope (Olympus FV 3000), followed by quantification using ImageJ (counting positive cells per HPF).

### 2.6 Cytokine profiling

Colon tissues were homogenized in RIPA buffer (1:10 w/v) using TissueLyser II (QIAGEN) at 25 Hz for 2 min and then centrifuged at 12,000 ×g for 15 min at 4 °C (Eppendorf 5424R, FA-45-30-11 rotor; Hamburg, Germany). The supernatants were assayed using ELISA to determine cytokine levels. Pro-inflammatory cytokines measured included TNF-α (R&D Systems, DY510; Minneapolis, MN, United States), IL-6 (R&D Systems DY506), and IL-1β (DY501, R&D Systems). Anti-inflammatory cytokines included IL-10 (R&D Systems, DY522) and IL-4 (DY504, R&D Systems). Protocols were followed according to the manufacturer’s instructions. Absorbance was measured at 450 nm using a Tecan microplate reader. Total protein concentration was quantified using the Bradford Assay (Bio-Rad #5000006) with BSA standards (0–2 mg/mL, 595 nm), and cytokine levels were normalized to protein content (pg/mg protein).

#### 2.6.1 Cell culture and treatment

RAW264.7 macrophages (purchased from ATCC, TIB-71; Manassas, VA, United States) were seeded at a density of 2 × 10^5 cells/well in 6-well plates (Corning, Cat. 3516; NY, United States) and cultured in complete DMEM (10% FBS and 1% penicillin/streptomycin) at 37 °C/5% CO_2_. Cells were divided into the following groups:

(1) Control: untreated;
(2) M1 model: lipopolysaccharides (LPSs) 100 ng/mL + interferon-γ (IFN-γ) 20 ng/mL for 24 h (Buchner, 2020);
(3) ZQG group: M1 model +10% ZQG-conditioned medium for 48 h. ZQG-conditioned medium was prepared by dissolving ZQG in saline (10 mg/mL), filtering it through 0.22 μm, and diluting it to 10% v/v in DMEM.
(4) PPAR-γ inhibition group: GW9662 (2-chloro-5-nitro-N-phenylbenzamide; MedChemExpress, HY-16578; Monmouth Junction, NJ, United States) was dissolved in DMSO (final concentration ≤0.1%) and administered at 10 μM for pretreatment for 1 h to inhibit PPAR-γ (Xu et al., 2025) before ZQG treatment.

#### 2.6.2 Flow cytometry

After treatment, cells were harvested using 0.25% trypsin–EDTA (Gibco, 25200056; Waltham, MA, United States), washed twice with PBS, and stained with the following antibodies for 30 min at 4 °C in the dark. Surface markers included anti-CD86-FITC (1:200, BD Biosciences, 553692) and anti-CD206-APC (1:200, BD Biosciences, 561153) in PBS containing 2% FBS. DAPI (1 μg/mL, Sigma, D9542) was added as a viability control to exclude dead cells. For gating, live cells were selected based on FSC-A/SSC-A profiles. Doublets were excluded using FSC-H/FSC-W. M1 (CD86^+^CD206^−^) and M2 (CD86^−^CD206^+^) populations were quantified as a percentage of total live cells (see Supplementary Figure S1 for gating hierarchy).

### 2.7 Statistical analysis

Data are presented as the mean ± SEM. A one-way ANOVA test was conducted, followed by Tukey’s post hoc test for multiple comparisons. Pearson’s correlation test was conducted for cytokine–pathway associations. *P* < 0.05 was considered significant (GraphPad Prism 9).

## 3 Results

### 3.1 ZQG attenuates DSS-induced colitis pathology

**FIGURE 1:**
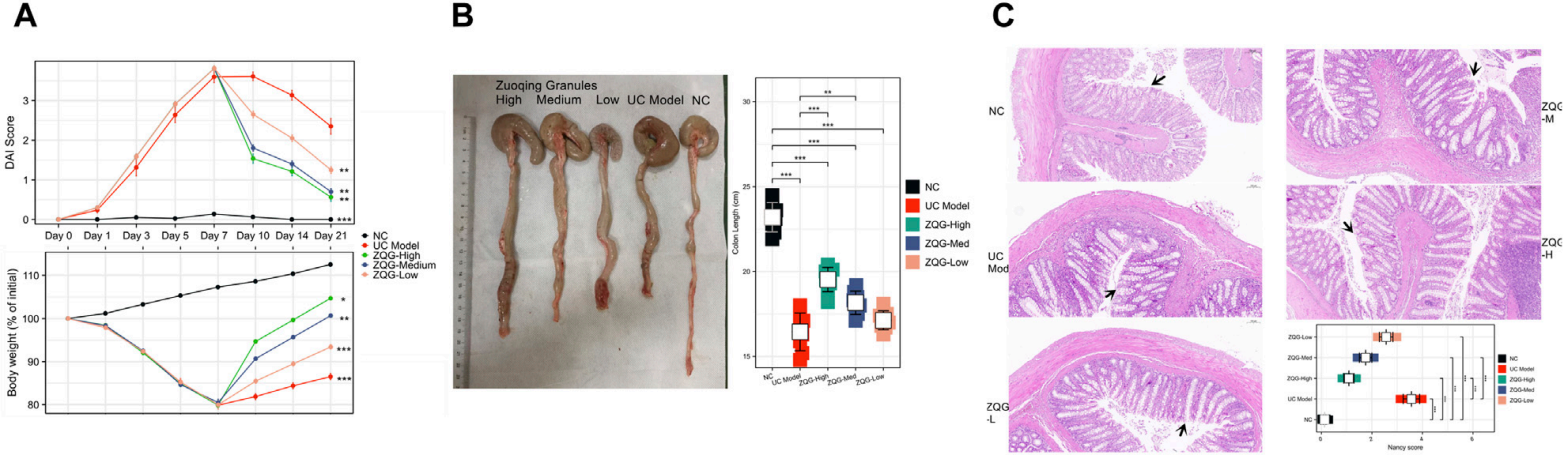
ZQG ameliorates DSS-induced colitis in rats. **(A)** DAI scores during the experimental timeline (n = 8/group). Data are presented as the mean ± SEM. **P < 0.01 vs. DSS model (one-way ANOVA followed by Tukey’s post hoc test). **(B)** Representative colon images (top) and quantification of colon length (bottom). Scale bar = 1 cm. **(C)** HE staining of colon tissues (×100 magnification) with Nancy histological scores. Black arrows indicate crypt damage. Scale bar = 100 μm.

In the DAI assessment (Figure 1A), DSS-treated rats exhibited significant weight loss, diarrhea, and rectal bleeding, with peak DAI scores reaching 3.8 ± 0.4 on Day 7. After ZQG treatment (from Day 8 onward), the DAI was dose-dependently reduced, with the high-dose group showing the most pronounced improvement (1.2 ± 0.3 on Day 14, *P* < 0.01 vs. DSS model).

In the analysis of colon length and macroscopic damage (Figure 1B), DSS caused severe colon shortening (16.7 ± 2.1 cm vs. NC 23.2 ± 1.8 cm, *P* < 0.001), which was ameliorated by ZQG (high dose: 19.6 ± 1.5 cm, *P* < 0.01).

Histological examination by HE staining (Figure 1C) showed that the UC model group exhibited crypt destruction, ulceration, and inflammatory infiltration (Nancy score: 3.5 ± 0.4). ZQG treatment preserved crypt architecture (high dose: 1.1 ± 0.2, *P* < 0.001).

### 3.2 ZQG modulates macrophage polarization

**FIGURE 2:**
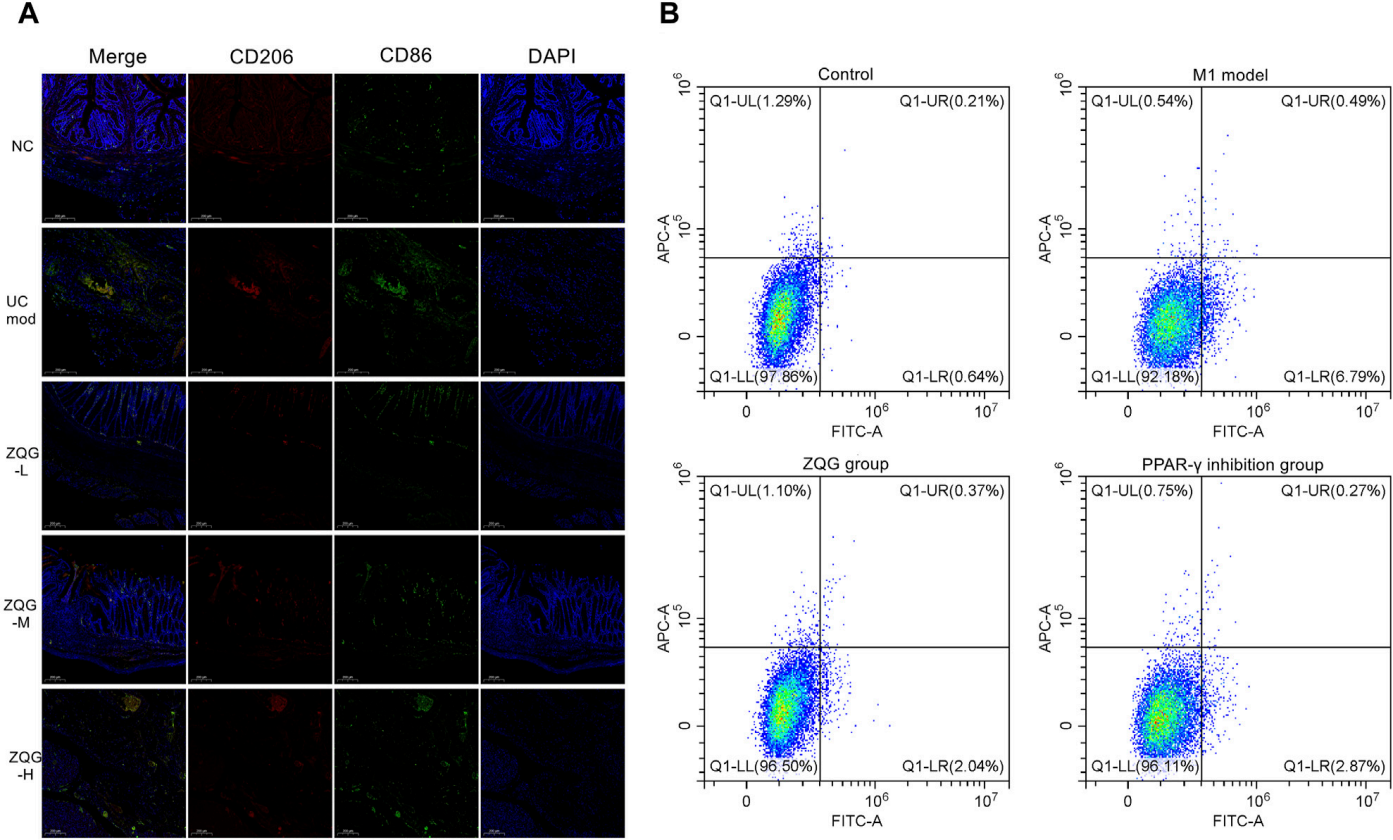
**(A)** ZQG modulates macrophage polarization in colonic tissues. Immunofluorescence staining of M1 (CD86^+^, green) and M2 (CD206^+^, red) macrophages (×400). Nuclei counterstained with DAPI (blue). Scale bar = 200 μm. Quantification (right panel): CD206^+^ cells: 26 ± 3 cells/HPF (ZQG-H) vs. 8 ± 1 cells/HPF (DSS), *P* < 0.001. CD86^+^ cells: 12 ± 2 cells/HPF (ZQG-H) vs. 28 ± 3 cells/HPF (DSS), *P* < 0.01. Data are presented as the mean ± SEM (n = 5 fields/group). Note: DAPI intensity variations may reflect DSS-induced nuclear fragmentation (UC model) and chromatin remodeling during ZQG-mediated repair (ZQG-H). **(B)** ZQG modulates macrophage polarization in RAW264.7 cells. Representative flow cytometry plots show M1 (CD86^+^) and M2 (CD206^+^) macrophage populations. Quadrants were defined using unstained and single-stained controls: Q1-LR (CD86^+^CD206^−^, M1) and Q1-UL (CD86^−^CD206^+^, M2). Quantification of CD86^+^ (M1) and CD206^+^ (M2) cells as a percentage of total live cells. Data are presented as the mean ± SEM (n = 8 independent experiments) (one-way ANOVA followed by Tukey’s test).

*In vivo*, immunofluorescence staining results (CD86^+^/CD206^+^) (Figure 2A) showed that DSS increased M1 macrophages (CD86^+^) (model: 28 ± 3 cells/HPF vs. NC: 8 ± 1 cells/HPF, *P* < 0.001), while ZQG reduced CD86^+^ cells (high dose: 12 ± 2 cells/HPF, *P* < 0.01). Conversely, ZQG enhanced M2 macrophages (CD206^+^) (high-dose: 26 ± 3 cells/HPF vs. model: 8 ± 1 cells/HPF, *P* < 0.001).

*In vitro*, to validate the direct effects of ZQG on macrophage polarization, flow cytometry analysis revealed that ZQG-conditioned medium significantly reduced M1 markers (CD86^+^ cells: 2.0% ± 0.1% vs. model 6.7% ± 0.3%, *P* < 0.01). PPAR-γ inhibition by GW9662 abolished ZQG’s effects (CD86^+^ cells: 2.87% ± 0.2% vs. ZQG alone 2.00% ± 0.1%, *P* < 0.01; CD206^+^ cells: 0.22% ± 0.05% vs. ZQG alone 0.37% ± 0.1%, *P* < 0.01) (Figure 2B).

### 3.3 ZQG regulates PPAR-γ/NF-κb/STAT1 signaling pathways

**FIGURE 3:**
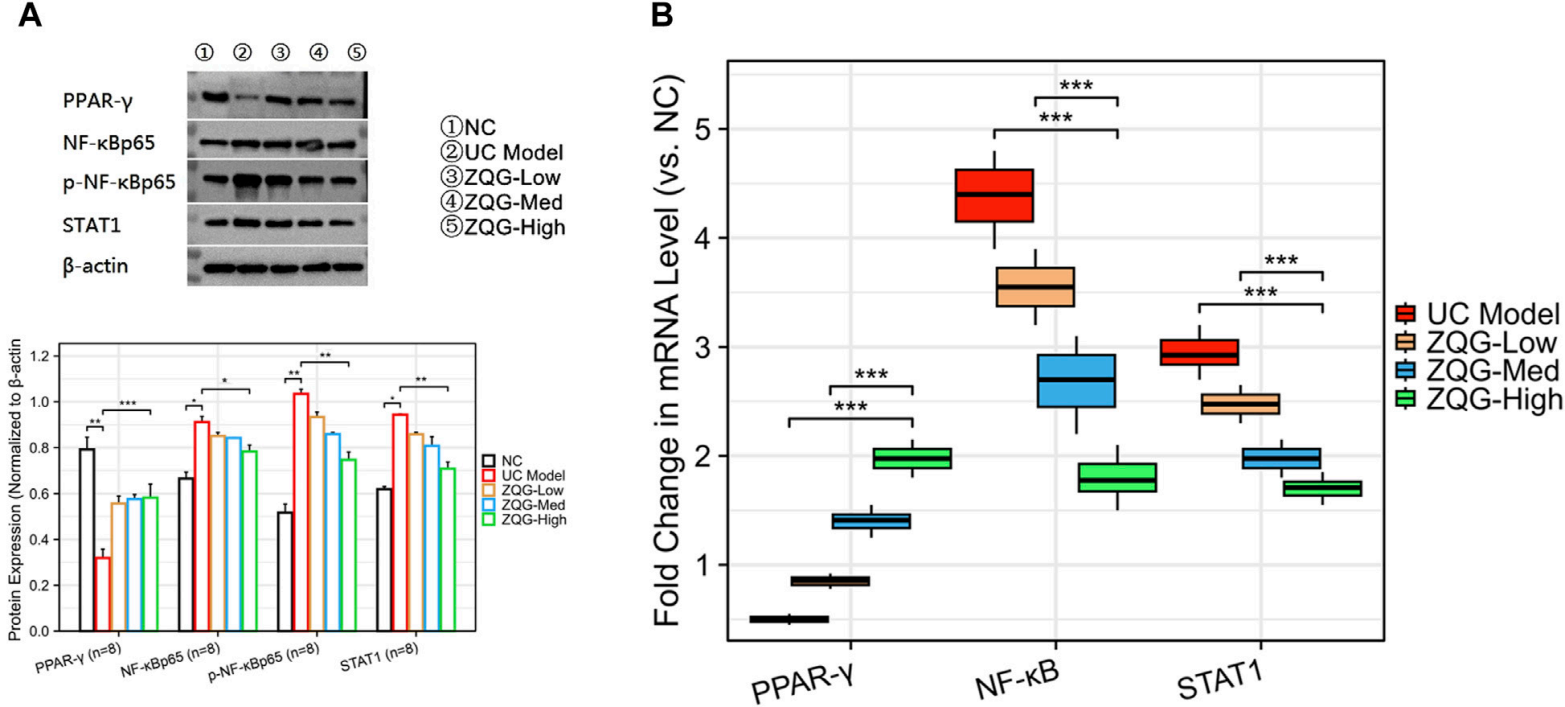
ZQG regulates PPAR-γ/NF-κB/STAT1 signaling pathways. **(A)** Western blot analysis of PPAR-γ, p-NF-κB p65, and STAT1 protein levels. β-Actin served as the loading control. **(B)** qPCR analysis of STAT1 and PPAR-γ mRNA expression normalized to β-actin (2^−ΔΔCT^ method). Data are presented as the mean ± SEM (n = 8). ***P < 0.01 vs. DSS model. Both qPCR and Western blot analyses used n = 8 independent biological replicates per group.

In Western blot analysis (Figure 3A), ZQG upregulated PPAR-γ expression (high dose: 2.1-fold increase, *P* < 0.01); ZQG inhibited p-NF-κB p65 (high dose: ↓43%, *P* < 0.01) and NF-κB p65 (high dose: ↓50%, *P* < 0.01); ZQG suppressed STAT1 activation (high dose: ↓48%, *P* < 0.01).

In qPCR validation (Figure 3B), STAT1 mRNA decreased (high dose: ↓40%, *P* < 0.05), while PPAR-γ mRNA increased (high dose: 2.0-fold, *P* < 0.01).

### 3.4 ZQG restores the cytokine balance in UC rats

**FIGURE 4:**
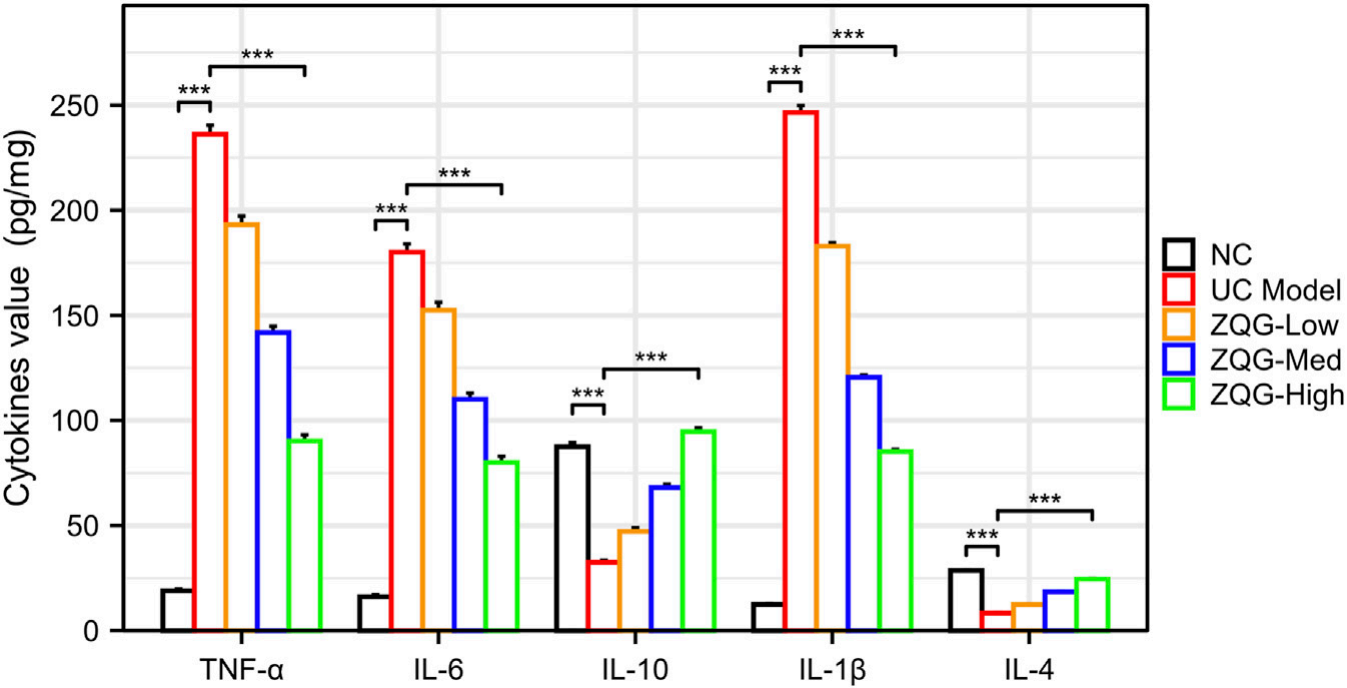
Cytokine concentrations in colon homogenates (pg/mg protein). ELISA quantification of TNF-α, IL-6, and IL-1β (M1-associated pro-inflammatory), along with IL-10 and IL-4 (M2-promoting anti-inflammatory cytokines) in colon homogenates. ***P < 0.001 vs. DSS model (n = 8). ***P < 0.001 vs. DSS model (n = 8).

ZQG treatment significantly (*p* < 0.001) modulated cytokine profiles in UC rats (Figure 4). The M1-associated pro-inflammatory cytokines were markedly reduced: TNF-α decreased from 235 ± 18 to 89 ± 12 pg/mg protein (62%), IL-6 from 180 ± 15 to 77 ± 10 pg/mg protein (57%), and IL-1β from 68 ± 5 to 26 ± 3 pg/mg protein (62%). Conversely, ZQG treatment substantially elevated M2-promoting anti-inflammatory cytokines: IL-10 increased from 32 ± 4 to 91 ± 8 pg/mg protein (185%) and IL-4 from 7.5 ± 0.8 to 23.2 ± 2.1 pg/mg protein (210%).

### 3.5 Correlation analysis

**FIGURE 5:**
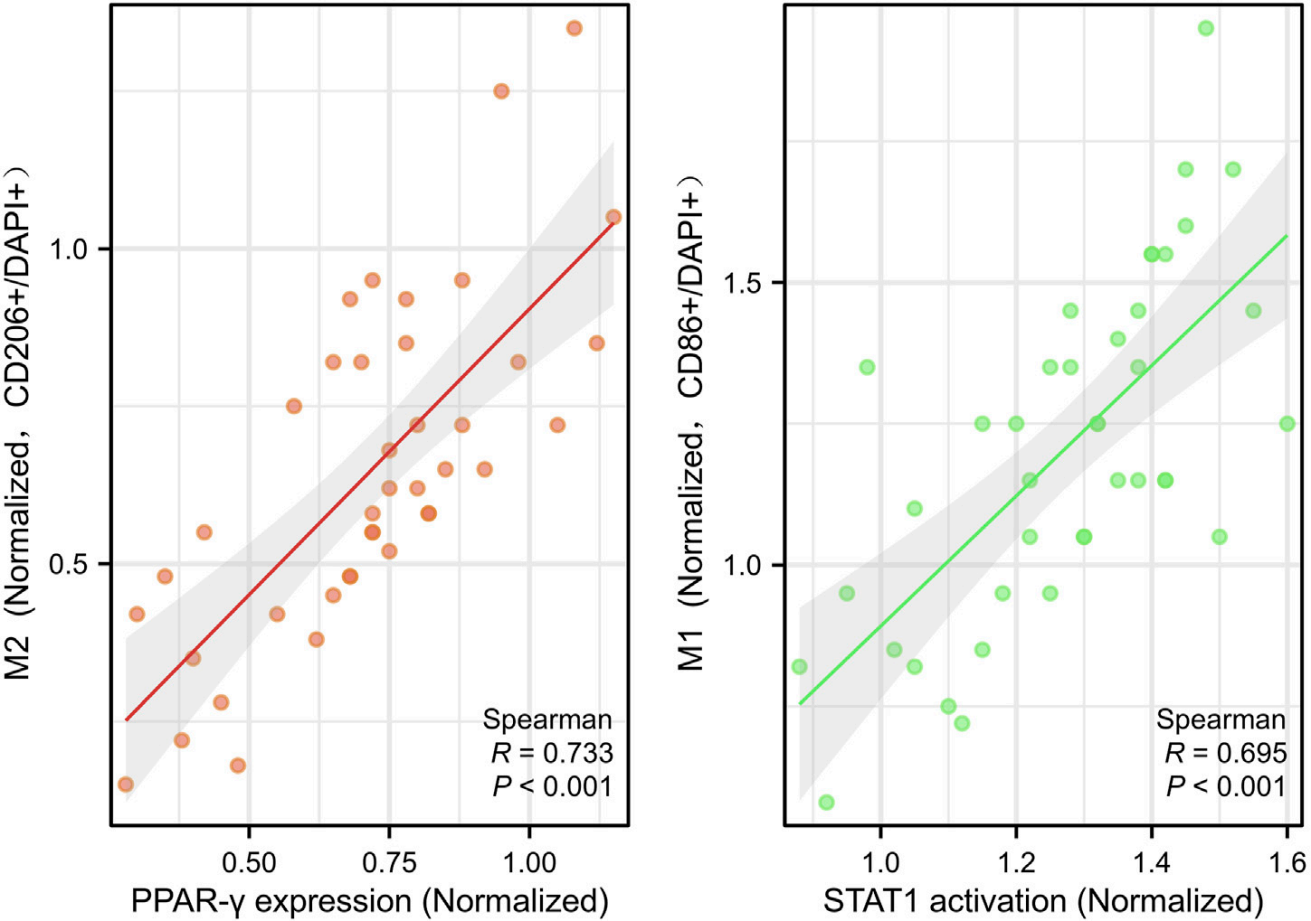
Correlation analysis of macrophage polarization markers with signaling molecules. Scatter plots with Pearson correlation coefficients. Left: PPAR-γ expression vs. M2 macrophage infiltration (CD206^+^ cells), r = 0.773, *P* < 0.001. Y-axes: PPAR-γ protein expression (OD/mm^2^, Western blot). Right: STAT1 activation vs. M1 macrophage polarization (CD86^+^ cells), r = 0.695, P < 0.001. Each point represents an individual rat (n = 40 total). Y-axes: STAT1 phosphorylation level (OD/mm^2^, Western blot).

Consistent with prior reports (Cheng et al., 2024; Zhang et al., 2024), PPAR-γ expression was positively correlated with M2 macrophage infiltration (r = 0.773, *P* < 0.001), while STAT1 activation was associated with M1 polarization (r = 0.695, *P* < 0.001) (Figure 5), further supporting their roles in ZQG-mediated macrophage reprogramming.

## 4 Discussion

Our data demonstrate that ZQG ameliorated colitis in a dose-dependent manner. Notably, the high-dose group reached the recovery threshold (disease activity index <1) on the 16th day, 1.2 days earlier than the medium-dose group, while the low-dose group and DSS model group failed to achieve this threshold during the observation period. This dose-dependent acceleration of recovery is inconsistent with spontaneous healing patterns, further supporting the pharmacological activity of ZQG. Our findings reveal three key mechanistic insights: (1) ZQG administration significantly shifts the M1/M2 macrophage balance toward an anti-inflammatory phenotype; (2) this effect is mediated through coordinated upregulation of PPAR-γ and suppression of NF-κB/STAT1 pathways; and (3) the macrophage polarization changes correlate with improved clinical outcomes and cytokine profiles.

The observed shift from M1 to M2 macrophage polarization represents a crucial mechanism underlying ZQG’s therapeutic effects. Our immunofluorescence data showing decreased CD86^+^ and increased CD206^+^ macrophages align with emerging evidence that rebalancing macrophage phenotypes can ameliorate colitis (Xiao et al., 2024). Notably, the strong positive correlations between PPAR-γ/M2 and STAT1/M1 macrophages have been documented in other contexts (He et al., 2021; Lin et al., 2024; Cao et al., 2023). Our study first establishes their significance in the context of ZQG treatment, demonstrating that this TCM formulation leverages conserved signaling axes to achieve therapeutic effects.

At the molecular level, our study identifies PPAR-γ as a critical mediator of ZQG’s effects. The 2.1-fold upregulation of PPAR-γ in high-dose ZQG groups corresponds with recent work demonstrating PPAR-γ’s role in promoting M2 polarization (Shao et al., 2023). The parallel 43%–50% reductions in p-NF-κB and NF-κB suggest that ZQG’s anti-inflammatory effects involve NF-κB pathway inhibition, consistent with known crosstalk between PPAR-γ and NF-κB signaling (Qin et al., 2024). STAT1 suppression (48% reduction) provides additional mechanistic insights as STAT1 is a well-established driver of M1 polarization (Figueroa-Valdes et al., 2025). Although qPCR and Western blot analyses consistently demonstrated PPAR-γ activation and NF-κB/STAT1 inhibition by ZQG, the magnitude of fold changes varied between the two techniques. This discrepancy may arise from methodological differences as qPCR detects mRNA levels with higher sensitivity, whereas Western blotting reflects post-translational protein dynamics. The reversal of ZQG’s effects by GW9662 in both systems strengthens the mechanistic link between PPAR-γ signaling and macrophage plasticity in UC therapy.

Cytokine profiling revealed significant reductions in M1-associated TNF-α (89%), IL-6 (57%), and IL-1β (62%), alongside markedly elevated M2-promoting IL-10 (185%) and IL-4 (210%). These coordinated changes provide direct functional evidence of ZQG-induced macrophage repolarization. These findings are clinically significant given the central role of TNF-α (Yang et al., 2025), IL-6 (El-Tanbouly and Abdelrahman, 2025), IL-10, and IL-1β/IL-4 (Wang F. et al., 2024) in UC pathogenesis and current anti-TNF biologic therapy (Jing et al., 2024).

Several limitations of this study should be noted. First, although our study focused on macrophage polarization, ZQG likely affects other immune cells in the colonic microenvironment. Second, the relative contributions of each botanical drug metabolite in the granule formulation warrant further investigation. Future studies should isolate individual metabolites to determine their specific mechanisms and potential synergistic effects.

## 5 Conclusion

Our results demonstrate that PPAR-γ/NF-κB/STAT1-mediated macrophage polarization is a central mechanism through which ZQG ameliorates experimental colitis, while acknowledging potential synergistic pathways warranting future investigation.

## Data Availability

The original contributions presented in the study are included in the article/Supplementary Material; further inquiries can be directed to the corresponding author.

## Abbreviations

UC, ulcerative colitis; ZQG, Zuoqing granule; DSS, dextran sulfate sodium; PPAR-γ, peroxisome proliferator-activated receptor gamma; NF-κB, nuclear factor kappa B; STAT1, signal transducer and activator of transcription 1; M1/M2, macrophage polarization phenotypes; TCM, traditional Chinese medicine; LPS lipopolysaccharide; IFN-γ, interferon-γ.
