# Supplementary figures and images for: Zuoqing granules attenuate ulcerative colitis via macrophage polarization modulation: involvement of the PPAR-γ/NF-κB/STAT1 signaling axis
Source: Front Pharmacol. 2025 Aug 11;16:1646545. doi: 10.3389/fphar.2025.1646545 (PMC12375609; doi:10.3389/fphar.2025.1646545)

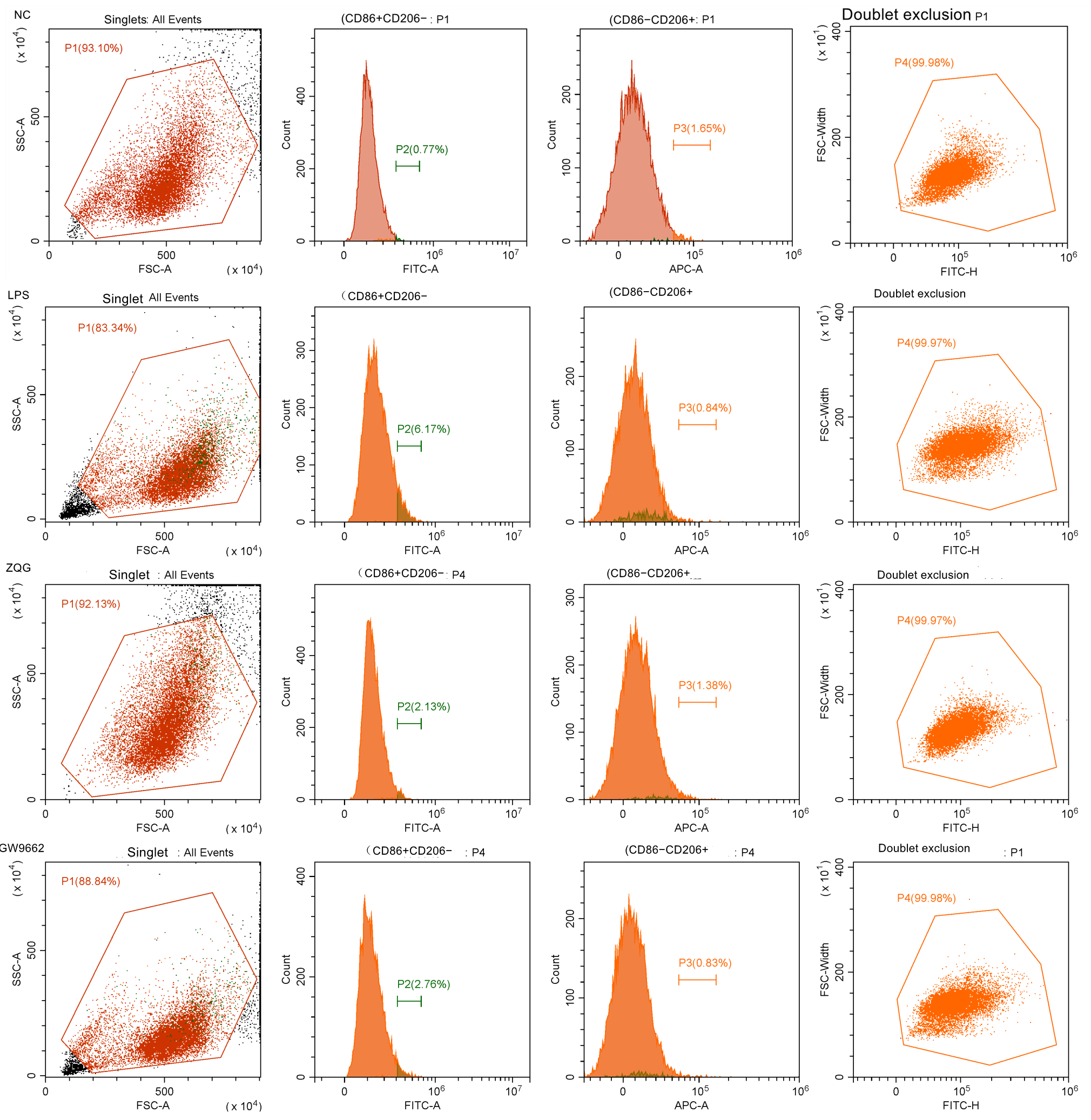

Supplement: Supplementary file 1 [file Image1.jpeg]
